# Characterization of the effects of outliers on ComBat harmonization for removing inter-site data heterogeneity in multisite neuroimaging studies

**DOI:** 10.3389/fnins.2023.1146175

**Published:** 2023-05-25

**Authors:** Qichao Han, Xiaoxiao Xiao, Sijia Wang, Wen Qin, Chunshui Yu, Meng Liang

**Affiliations:** ^1^School of Medical Technology, School of Medical Imaging, Tianjin Key Laboratory of Functional Imaging, Tianjin Medical University, Tianjin, China; ^2^Department of Radiology and Tianjin Key Laboratory of Functional Imaging, Tianjin Medical University General Hospital, Tianjin, China

**Keywords:** outliers, site effect, ComBat harmonization, imaging derived phenotypes, magnetic resonance imaging, multisite

## Abstract

Data harmonization is a key step widely used in multisite neuroimaging studies to remove inter-site heterogeneity of data distribution. However, data harmonization may even introduce additional inter-site differences in neuroimaging data if outliers are present in the data of one or more sites. It remains unclear how the presence of outliers could affect the effectiveness of data harmonization and consequently the results of analyses using harmonized data. To address this question, we generated a normal simulation dataset without outliers and a series of simulation datasets with outliers of varying properties (e.g., outlier location, outlier quantity, and outlier score) based on a real large-sample neuroimaging dataset. We first verified the effectiveness of the most commonly used ComBat harmonization method in the removal of inter-site heterogeneity using the normal simulation data, and then characterized the effects of outliers on the effectiveness of ComBat harmonization and on the results of association analyses between brain imaging-derived phenotypes and a simulated behavioral variable using the simulation datasets with outliers. We found that, although ComBat harmonization effectively removed the inter-site heterogeneity in multisite data and consequently improved the detection of the true brain-behavior relationships, the presence of outliers could damage severely the effectiveness of ComBat harmonization in the removal of data heterogeneity or even introduce extra heterogeneity in the data. Moreover, we found that the effects of outliers on the improvement of the detection of brain-behavior associations by ComBat harmonization were dependent on how such associations were assessed (i.e., by Pearson correlation or Spearman correlation), and on the outlier location, quantity, and outlier score. These findings help us better understand the influences of outliers on data harmonization and highlight the importance of detecting and removing outliers prior to data harmonization in multisite neuroimaging studies.

## 1. Introduction

With the rapid development of neuroimaging techniques and information technology, the utilization of big data is becoming a trend in neuroimaging studies in recent years for several reasons (Poline et al., 2012). First, large sample sizes provide higher statistical power and are more likely to produce reliable results. Second, large sample sizes are more representative of populations and thus the results are more generalizable and have more significant implications in real life (Zuo et al., 2014; Marek et al., 2022). Third, a sample size of several thousands or even tens of thousands is often mandatory in some newly emerging research fields to address important questions concerning small effects, such as genome-wide association studies (GWAS) or exposome-wide association studies (EWAS) of imaging-derived phenotypes (IDPs; Stein et al., 2012; Hibar et al., 2015; Mulugeta et al., 2022), or studies of rare diseases (Patrick et al., 2022), developmental population neuroscience (Zuo et al., 2018), and statistical connectomics (Chung et al., 2021).

Using data from multiple sites is often inevitable to form a large sample size for neuroimaging studies. For example, almost all currently existing big neuroimaging datasets, including Adolescent Brain Cognitive Development (ABCD; Casey et al., 2018), Enhancing NeuroImaging Genetics Through Meta-Analysis (ENIGMA; Stein et al., 2012), Imaging Genetics (IMAGEN; Schumann et al., 2010), UK Biobank (UKBB; Littlejohns et al., 2020), Alzheimer’s Disease Neuroimaging Initiative (ADNI; Mueller et al., 2005), and Chinese Imaging Genetics (CHIMGEN; Xu et al., 2020), are composed of cohorts from multiple sites.

However, due to the differences in scanning equipment and techniques, multisite datasets often present differences in the distribution of data collected from different sites, also known as site effect or inter-site heterogeneity of data. The problem of inter-site heterogeneity in multisite studies needs to be attended to carefully as it is an important confounding factor for statistical analyses and will lead to unreliable results if mishandled or disregarded (Takao et al., 2011; Zhu et al., 2011; Shinohara et al., 2017). Several data harmonization methods have been proposed to correct the inter-site heterogeneity, such as ComBat (Fortin et al., 2017), LICA (Groves et al., 2011), RAVEL (Fortin et al., 2016), RISH (Mirzaalian et al., 2018), and Neuroharmony (Garcia-Dias et al., 2020). Among these methods, ComBat (combating batch effects) has the advantages of estimating both additive and multiplicative site effects and is capable of preserving biological variances of interests while removing inter-site heterogeneity, and thus is the most commonly used method in the field of neuroimaging, including the harmonization of structural MRI data, functional MRI data, and diffusion tensor imaging (DTI) data (Fortin et al., 2017, 2018; Yu et al., 2018).

Another important issue in multisite neuroimaging data with large sample sizes is the prevalence of outliers, defined as observations that dramatically deviate from the majority of the data (Tan et al., 2006). There are mainly two sources of outliers in neuroimaging data. The first is imaging artifacts or noises, such as magnetic field inhomogeneity or head movements during scanning, resulting in inaccurate morphometric measures of the brain and consequently outliers (Van Dijk et al., 2012; Reuter et al., 2015). Second, some outliers may represent genuine abnormalities of the brain due to diseases; for example, a very small volume of the limbic regions may signal a risk of Alzheimer’s disease (Wang et al., 2015). Regardless of the sources, these outliers could impose detrimental effects on the results of statistical analyses and should be handled with care during data analyses.

The issue caused by the presence of outliers in data analyses becomes more complicated in multisite neuroimaging studies due to the inter-site heterogeneity in the data. It is likely that outliers may also bias the results of data harmonization for the removal of inter-site heterogeneity and even exacerbate the heterogeneity of multisite data, thus leading to unexpectedly erroneous results. Therefore, outlier removal has been considered as a regular step of the standard workflow of data harmonization. Nonetheless, it is worth noting that ComBat has been suggested to be more robust to outliers even in small sample sizes compared with other data harmonization methods such as singular-value decomposition (Alter et al., 2000), distance weighted discrimination (Benito et al., 2004), and location and scale model (Li and Wong, 2003). So far, there has not been any systematic investigation of whether and how the presence of outliers would affect the effectiveness of ComBat harmonization in multisite neuroimaging studies.

To address this question, in the present study we generated a series of multisite simulation datasets with or without outliers based on a real large neuroimaging dataset (CHIMGEN; Xu et al., 2020). We first used the simulation dataset without outliers to verify the effectiveness of ComBat harmonization in the removal of inter-site heterogeneity. We then used the simulation datasets with outliers to test whether the presence of outliers could reduce the effectiveness of ComBat harmonization and to characterize how such detrimental effects of outliers on ComBat harmonization effectiveness were dependent on outlier properties such as their location (i.e., where the outliers are, e.g., which sites, unilateral or bilateral), quantity (i.e., how many outliers are present in the data), and deviation level (measured by outlier score, representing how far the outliers deviate from the majority of the data). Importantly, the assessments of the effectiveness of ComBat harmonization and the detrimental effects of outliers were not only performed on the multisite data distributions but also on the results of association analyses between brain imaging measures and behavioral variables using the simulated multisite data. The reason why we tested the effects of outliers on the effectiveness of ComBat harmonization using simulated data rather than real data was that the location, quantity, and deviation of the outliers were very limited in this real dataset whereas the simulated datasets with varying outlier location, quantity, and deviation level could allow us to better characterize the effects of outliers in various outlier scenarios.

## 2. Materials and methods

### 2.1. Generation of a normal simulation dataset

A normal simulation dataset (i.e., a simulation dataset without outliers) was generated according to the statistical properties (mean and covariance) of a real brain imaging dataset – the grey matter volume (GMV) computed from the T1 weighted imaging data of the CHIMGEN database (Xu et al., 2020). The detailed procedure is as follows: First, we selected the brain imaging data from three sites (sample size =1743, 804, and 466 for Site 1, Site 2, and Site 3, respectively; 3,013 samples in total) of the CHIMGEN database, which showed differences in data distribution between sites. Second, the images of whole brain were parcellated into 273 brain regions according to the combined Brainnetome atlas including both cerebrum and cerebellum (Fan et al., 2016; available from http://atlas.brainnetome.org/download.html). It should be noted that the original combined Brainnetome atlas contains 274 regions; however, one region was reduced to 0 voxels after downsampling the original atlas image (1.25 × 1.25 × 1.25 mm^3^ voxel size) to match with our current data (1.5 × 1.5 × 1.5 mm^3^ voxel size), and thus 273 regions in total remained for subsequent analyses. Third, we obtained a GMV for each brain region and subject, resulting in a dataset consisting of 273 IDPs and 3,013 subjects in total. Fourth, for each site, outliers were detected and removed from the data for each IDP and consequently 2,894 samples in total (1731 in Site 1, 804 in Site 2, and 359 in Site 3) remained for subsequent analyses. Here, outliers were defined as the values that were at least three times the interquartile range (IQR) below the lower quartile (Q1) or above the upper quartile (Q3) of the data distribution, as suggested by Tukey in 1977 (Tukey, 1977). Fifth, we calculated the mean vectors and the covariance matrix of the 273 IDPs for each of the three sites. Finally, based on the mean vectors and the covariance matrices computed from the real data of the three sites, we then used the MATLAB function *mvnrnd* to generate three sets of random values (1,000 subjects and 273 IDPs for each subject in each set) following the normal distribution with the same mean and covariance as the real brain imaging dataset. The generated three sets of random values composed the simulated brain imaging data of three sites, with 1,000 samples in each site.

To generate a simulated behavioral variable that would be correlated with some of the 273 simulated IDPs, we selected the 187^th^ IDP (IDP-187) as a representative IDP (because the IDP-187 showed correlations with many other IDPs), performed z-score normalization ([IDP-mean(IDP)]/std.(IDP)), and then added some Gaussian noises (mean = 0, variance = 0.04). The resultant variable served as a simulated behavioral variable without inter-site heterogeneity. In this way, this simulated behavioral variable would correlate with the IDP-187 as well as some other IDPs showing correlations with the IDP-187. This was performed for each site to generate the corresponding simulated behavioral variable for each site. Therefore, we generated 273 simulated IDPs and 1 simulated behavioral variable for each site.

### 2.2. Generation of the simulation data with outliers

To generate the simulation datasets with outliers, we added some extra samples with extreme values (i.e., outliers) to the above generated normal simulation dataset. As aforementioned, in the present study, outliers were defined as the values that were at least three times the IQR below the lower quartile Q1 or above the upper quartile Q3. We further quantified the deviation of outliers for each IDP using outlier scores. Specifically, outlier scores were defined using the following Eq. (1):


(1) $$Outlierscore=\frac{\min\left(\left|IDP-Q1\right|,\left|IDP-Q3\right|\right)}{IQR}$$

where Q1 denotes the lower quartile, Q3 denotes the upper quartile, and IQR denotes interquartile. To generate simulated outliers for a given site, we used the MATLAB function *mvnrnd* with a mean vector of [Q3 + Outlier score × IQR] (if the outlier was added to the upper side of the data distribution) or [Q1-Outlier score × IQR] (if the outlier was added to the lower side of the data distribution) and a covariance matrix which was 0.003^2^ times the correlation matrix of the normal real data. The simulated outliers were then added to the corresponding site of the normal simulation data. Here, we chose a relatively small standard deviation (i.e., 0.003) for generating outliers so that we could easily control the outlier scores.

To test how the presence of outliers in different scenarios affect the effectiveness of ComBat harmonization, the outliers were added to the normal simulation dataset in six different ways which varied in quantity (i.e., how many outliers were present), outlier score (i.e., how far the outliers were deviated from the “normal” data), and location of outliers (i.e., outliers on only one side or both sides of the data median in only one site or two sites): (A) Scenario “one-site unilateral”: outliers were added to the upper side of Q3 in Site 1; (B) Scenario “one-site bilateral”: the same number of outliers with the same outlier scores were added to both sides of the data distribution (i.e., the upper side of Q3 and the lower side of Q1) in Site 1; (C) Scenario “one-site different”: the same number of outliers were added to both sides of the data distribution (i.e., the upper side of Q3 and the lower side of Q1) but with different outlier scores (the outlier score on the upper side of Q3 was fixed to 40, but the outlier score on the lower side varied in seven different values: 3, 5, 10, 15, 20, 30, and 40) in Site 1; (D) Scenario “two-site unilateral”: the same number of outliers with the same outlier scores were added to the upper side of Q3 in both Sites 1 and 2; (E) Scenario “two-site bilateral”: the same number of outliers with the same outlier scores were added to the lower side of Q1 and the upper side of Q3 in both Sites 1 and 2; (F) Scenario “two-site different”: the same number of outliers were added to the upper side of Q3 in Site 1 and the lower side of Q1 in Site 2 (i.e., the outliers were added to different sides in different sites) but with the same outlier scores. For each of the six scenarios, the quantity of the added outliers varied in five different values (0.2, 0.6, 1, 5, and 10% of the sample size of the normal simulation dataset) and the outlier scores varied in seven different values (3, 5, 10, 15, 20, 30, and 40). Note that, to maintain the same sample size in all sites, for the first three scenarios (i.e., all three “one-site” scenarios) an equal number of normal values as the total number of the outliers added to Site 1 were also added to Site 2 and 3, and for the last three scenarios (i.e., all three “two-site” scenarios) an equal number of normal values as the number of the outliers added to Site 1/2 were also added to Site 3. Then, for each site, an equal number of simulated values (generated using the same approach described in Section 2.1) were also added to the simulated behavioral variable to make sure that there were equal number of samples for the simulated IDPs and behavioral variable. Note that, no outliers, but only normal values, were added to the simulated behavioral data.

### 2.3. ComBat harmonization

ComBat was initially developed to eliminate batch effects in microarray gene expression data (Johnson et al., 2007) and is now widely used to correct site effects of multisite neuroimaging data (Fortin et al., 2017, 2018; Yu et al., 2018). The ComBat algorithm is an empirical Bayesian-based method for removing unwanted variations associated with the scanning sites or equipment while retaining biologically relevant information of interest in the data, such as age, gender, or drug effects.

Taking voxel-wise grey matter volume (GMV) for example, ComBat algorithm models the raw GMV using the following Eq. (2):


(2) $$Y_{ijv}=\alpha_{v}+X_{ij}\beta_{v}+\gamma_{iv}^{\prime }+\delta_{iv}\varepsilon_{i\dot{j}v}$$

where $Y_{ijv}$ denotes the raw GMV for Site *i*, Subject *j*, and Voxel *v*, $\alpha_{v}$ denotes the average GMV of all subjects in all sites for Voxel *v*, $X_{ij}$ denotes a design matrix for the covariates of interest (e.g., age) and $\beta_{v}$ denotes a vector of regression coefficients corresponding to $X_{ij}$ for Voxel *v*, $\gamma_{iv}^{\prime }$ and $\delta_{iv}$ denote the additive and multiplicative site effect of Site *i* for Voxel *v*, respectively, and $\varepsilon_{i\dot{j}v}$ denotes the error term that follows a normal distribution with mean zero and variance $\sigma_{v}^{2}$ .

As the overall differences in GMV between different voxels may lead to errors in the estimation of the prior distribution of site effect, the ComBat algorithm first standardizes the data to have comparable overall mean and variance across different voxels. The equation for the standardization is


(3) $$Z_{ijv}=\frac{Y_{ijv}{\hat{\alpha }}_{v}-X_{ij}{\hat{\beta }}_{v}}{{\hat{\sigma }}_{v}}$$

$Z_{ijv}$ denotes the standardized GMV for Site *i*, Subject *j*, and Voxel *v*. ${\hat{\alpha }}_{v}$ , ${\hat{\beta }}_{v}$ , and ${\hat{\sigma }}_{v}$ are the estimates of $\alpha_{v}$ , $\beta_{v}$ , and $\sigma_{v}$ . Assuming the standardized data $Z_{ijv}\sim N\left(\gamma_{iv},\delta_{iv}^{2}\right)$ , in which the site effect parameters are assumed to have the parametric prior distributions: $\gamma_{iv}\sim N\left(\gamma_{i},\tau_{i}^{2}\right)$ , $\delta_{iv}^{2}\sim InverseGamma\left(\lambda_{i},\theta_{i}\right)$ ComBat algorithm subsequently estimates the site effect parameters $\gamma_{iv}^{\prime }$ and $\delta_{iv}$ using conditional posterior means, and the corresponding estimates are denoted as $\gamma_{iv}^{*}$ and $\delta_{iv}^{*}$ .

The data are eventually adjusted using Eq. (4), with $Z_{ijv}^{ComBat}$ representing the data with site effect removed:


(4) $$Z_{ijv}^{ComBat}=\frac{Z_{ijv}-{\hat{\alpha }}_{v}-X_{ij}{\hat{\beta }}_{v}-\gamma_{iv}^{*}}{\delta_{iv}^{*}}+{\hat{\alpha }}_{v}+X_{ij}{\hat{\beta }}_{v}$$

### 2.4. Assessing the effectiveness of ComBat harmonization

For the normal simulation data, heterogeneity among sites was evaluated in three ways (1) We plotted a density curve of all IDP values for each subject to show differences in IDP values across sites, (2) To visualize the IDP data distribution of all subjects for the three sites using a scatter plot in a two-dimensional space, we reduced the dimensionality of the IDP data (273 IDPs) to the first two principal components (PCs) using principal components analysis (PCA), (3) To test whether some significant IDP-behavior correlations that were not detected due to data heterogeneity among sites could be recovered after ComBat harmonization, we calculated the Pearson correlation coefficient (*r*) between each IDP and the behavioral variable before and after ComBat harmonization using the subjects within each site (i.e., within-site correlation to avoid the effect of inter-site heterogeneity) and using the subjects across all sites (i.e., across-site correlation, denoted as a combined site, to test the effect of inter-site heterogeneity). We plotted these IDP-behavior correlation coefficients as violin-box plots and also calculated the intraclass correlation coefficients (ICCs; Shrout and Fleiss, 1979; Shrout, 1998) of the IDP-behavior correlation coefficients between each pair of single sites and between each single site and the combined site to assess the consistency of the IDP-behavior correlation coefficients between sites before and after ComBat harmonization. Generally, ICC ≥ 0.80 were considered as high consistency, 0.80 > ICC ≥ 0.60 as moderate consistency, 0.60 > ICC ≥ 0.40 as good consistency, 0.40 > ICC ≥ 0.10 as poor consistency, and ICC < 0.1 as no consistency. We also assessed the number of high IDP-behavior correlations (*r* ≥ 0.6) for each single site and the combined site.

### 2.5. Assessing the impact of outliers on the effectiveness of ComBat harmonization

Based on the normal simulation dataset and the simulation datasets with outliers, the impact of outliers on the effectiveness of removal of inter-site heterogeneity by the ComBat harmonization algorithm was evaluated in two ways: (1) whether the site effects were successfully removed after ComBat harmonization when outliers were present in the data, and (2) whether the estimation of the associations between IDPs and behavioral variables were affected by the presence of outliers after ComBat harmonization. For the evaluation of (1), we visualized the IDP values of all data samples of all sites using the first and the second PCs of PCA under all six outlier scenarios (i.e., “one-site unilateral,” “one-site bilateral,” “one-site different,” “two-site unilateral,” “two-site bilateral,” “two-site different,” as described in section 2.2), and with varying outlier quantities and outlier scores, before and after ComBat harmonization. We also calculated the mean and variance of a representative IDP (IDP-187) of normal samples under all outlier quantities and outlier scores. As for the evaluation of (2), we assessed the changes in the correlation coefficients between IDPs and behavioral variables (measured by both Pearson correlation coefficient *r* and Spearman rank correlation coefficient *ρ*) before and after ComBat harmonization under all six outlier scenarios compared with those where outliers were absent. Furthermore, to quantify the effect of outlier location (i.e., outliers present in one or two sites, unilaterally or bilaterally) on the IDP-behavior relationships, we calculated the ICCs of the 35 IDP-behavior correlation coefficients (5 outlier quantities and 7 outlier scores, thus 35 in total) among the six outlier scenarios for each site (within-site correlations) and the combined site (across-site correlations).

## 3. Results

### 3.1. Effectiveness of ComBat harmonization

**Figure 1:**
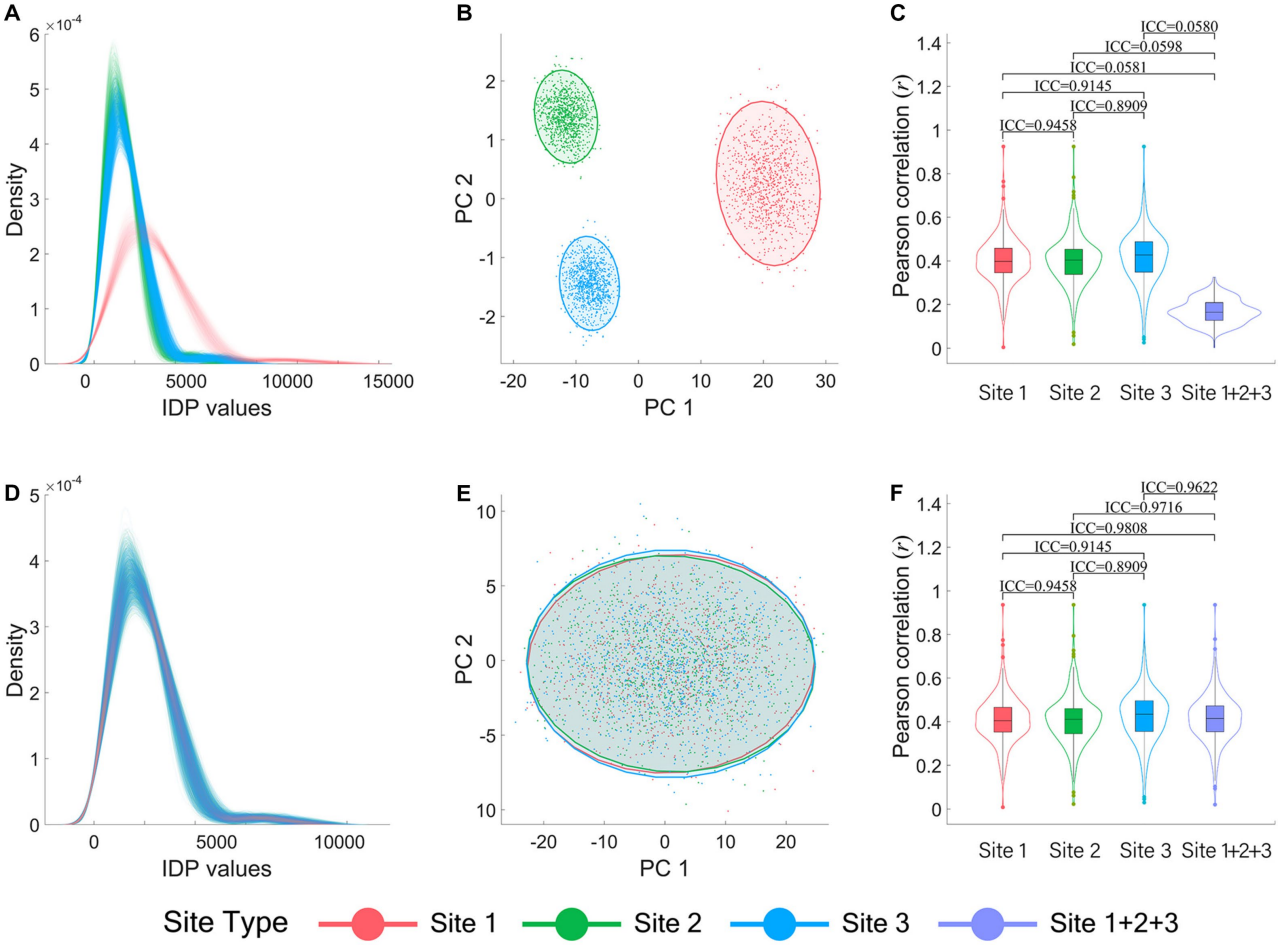
Visualization of data heterogeneity between sites in the normal simulation dataset before and after ComBat harmonization. **(A-C)** are the results before ComBat harmonization, and **(D-F)** are the results after ComBat harmonization. **(A)** and **(D)** show the IDP data density curves of each subject. **(B)** and **(E)** show the IDP data scatter plots presented in two dimensions of their first and second PCs. **(C)** and **(F)** show the violin-box plots of Pearson correlation coefficients (*r*) between the 273 IDPs and the behavioral variable. All the plots are color-coded by sites, with red color representing Site 1, green representing Site 2, blue representing Site 3, and purple representing the combined site (i.e., Site 1 + 2 + 3). PC: principal component.

Figures 1A-C illustrate the inter-site differences in the simulated brain imaging data of the normal simulation dataset before ComBat harmonization. Data density curves of all subjects, categorized by sites (indicated by different colors), are presented in Figure 1A. It demonstrates substantial inter-site differences which are much larger than the data variance within sites. The inter-site heterogeneity is also clearly evident in the two-dimensional (2-D) data visualization of all subjects from different sites using the first two PCs (Figure 1B). Furthermore, as presented in the violin-box plots depicting the distribution of the Pearson correlation coefficients (*r*) between all IDPs and the behavioral variable of each site (Site 1, 2, 3) and the combined site (Site 1 + 2 + 3) in Figure 1C, the IDP-behavior across-sites correlations became much weaker (*r* ≈ 0.2) compared with the within-site correlations (*r* ≈ 0.4). The ICC values of the IDP-behavior correlation coefficients between different sites and between each site and the combined site also showed that the IDP-behavior correlation coefficients changed greatly (ICCs ranged between 0.0580 and 0.0598) when calculated across subjects of different sites (i.e., the combined site) compared with those when calculated across subjects within sites (ICCs ranged between 0.8909 and 0.9458). Moreover, 13 high IDP-behavior correlations (*r* ≥ 0.6) were identified in all three sites for the within-site correlations, but none reached 0.60 for the across-site correlations.

Figures 1D-F illustrate the corresponding results after ComBat harmonization in terms of the data density curve plots (Figure 1D), the 2-D data visualization using the first two PCs (Figure 1E), and the violin-box plots of the IDP-behavior correlation coefficients (Figure 1F). As shown in Figures 1D,E, the data distributions were largely overlapped and the data heterogeneity among sites was no longer observable after ComBat harmonization. Moreover, after ComBat harmonization, the IDP-behavior correlation coefficients obtained within each single site remained almost identical with before ComBat harmonization, but those obtained across different sites (Site 1 + 2 + 3) were improved greatly and at the same level as within-site correlation coefficients (Figure 1F). The ICCs of the IDP-behavior correlation coefficients between different single sites ranged between 0.8909 and 0.9458, and the ICCs between each single site and the combined site ranged between 0.9622 and 0.9808 (Figure 1F). Notably, 14 high IDP-behavior correlations (*r* ≥ 0.6) were identified for the across-site correlations after ComBat harmonization which included all 13 high IDP-behavior correlations (*r* ≥ 0.6) identified in all three sites for the within-site correlations.

### 3.2. Impact of outliers on the effectiveness of ComBat harmonization

**Figure 2:**
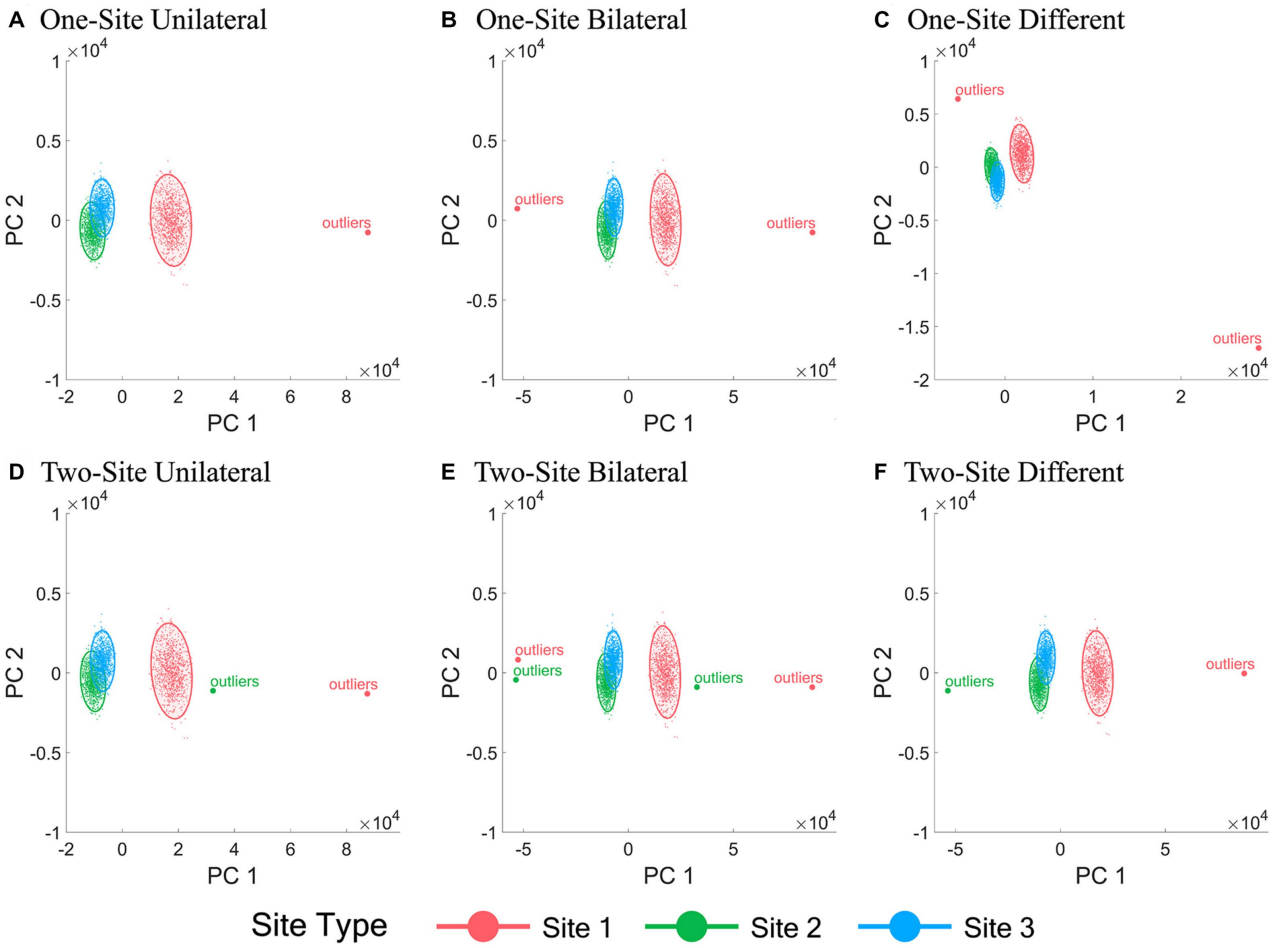
Scatter plots of the first two principal components of all IDPs before ComBat harmonization. **(A-F)** represent the six outlier scenarios described in Section 2.2 with a fixed outlier quantity = 1% of the normal simulation data sample size and outlier score = 10. The principal components (PCs) were obtained by performing principal component analysis of all IDP values of all subjects from all sites before ComBat harmonization. Data samples from Sites 1, 2, and 3 are colored by red, green, and blue, respectively. Each dot represents a data sample (i.e., a subject).

**Figure 3:**
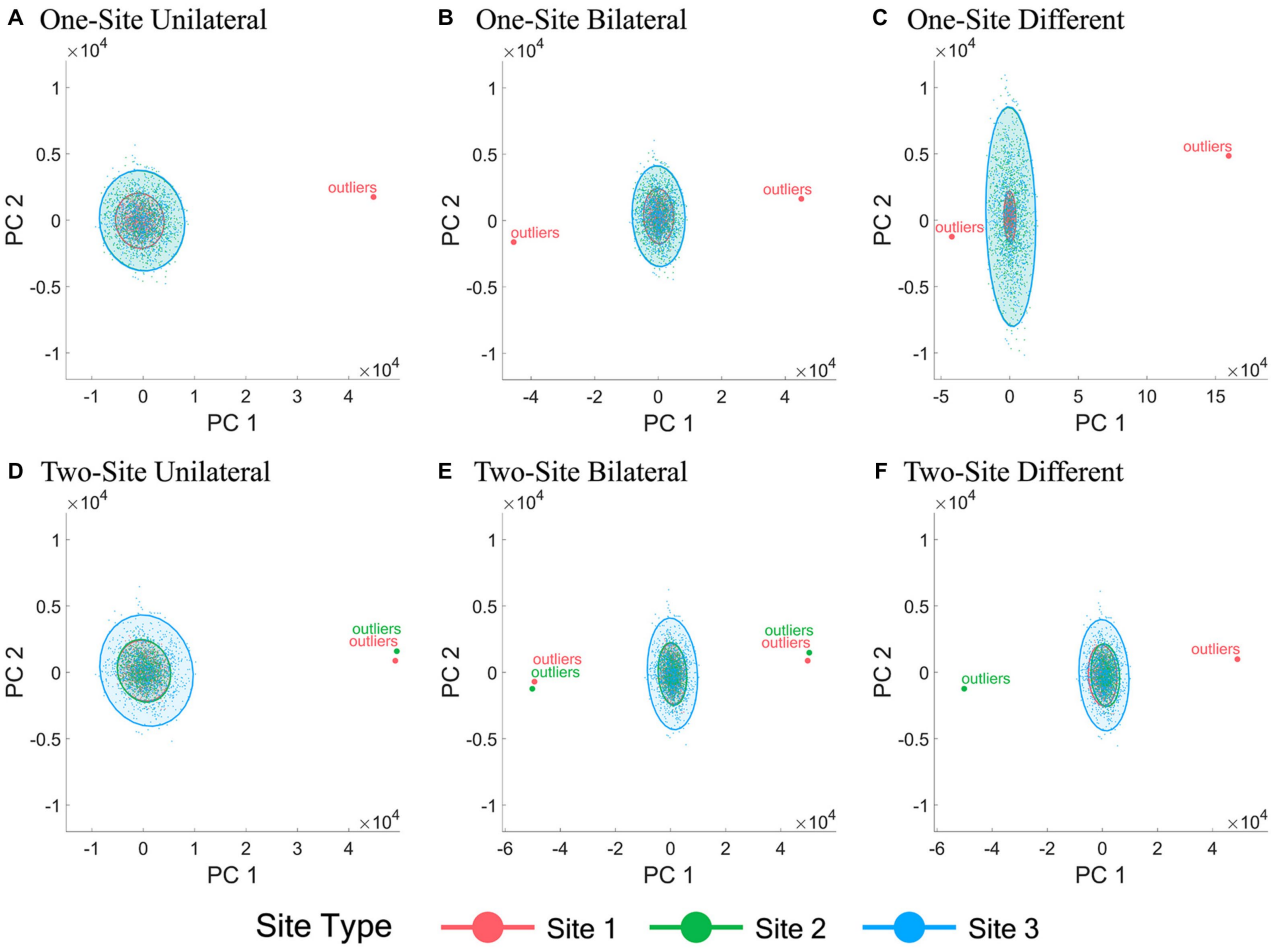
Scatter plots of the first two principal components of all IDPs after ComBat harmonization. **(A-F)** represent the six outlier scenarios described in Section 2.2 with a fixed outlier quantity = 1% of the normal simulation data sample size and outlier score = 10. The principal components (PCs) were obtained by performing principal component analysis of all IDP values of all subjects from all sites after ComBat harmonization. Data samples from Sites 1, 2, and 3 are colored by red, green, and blue, respectively. Each dot represents a data sample (i.e., a subject).

The 2-D data visualization of all subjects from different sites using the first two PCs of all IDPs under all six outlier scenarios with a fixed outlier quantity (1% of the normal simulation data sample size) and outlier score (10) before and after ComBat harmonization are shown in Figures 2, 3, respectively. Figure 2 demonstrated the inter-site heterogeneity in the data distribution (both the mean and the variance) in the presence of outliers before ComBat harmonization. Compared with Figures 2, 3 revealed that, under the condition of outlier quantity = 1% and outlier score = 10, the centers of the data distributions of the three sites were largely aligned after ComBat harmonization. However, Figure 3 also clearly showed that the data distributions became much denser for the sites with outliers (Site 1 in panels A-C, and Sites 1&2 in panels D-F) compared with those without outliers (Sites 2&3 in panels A-C, and Site 3 in panels D-F).

**Figure 4:**
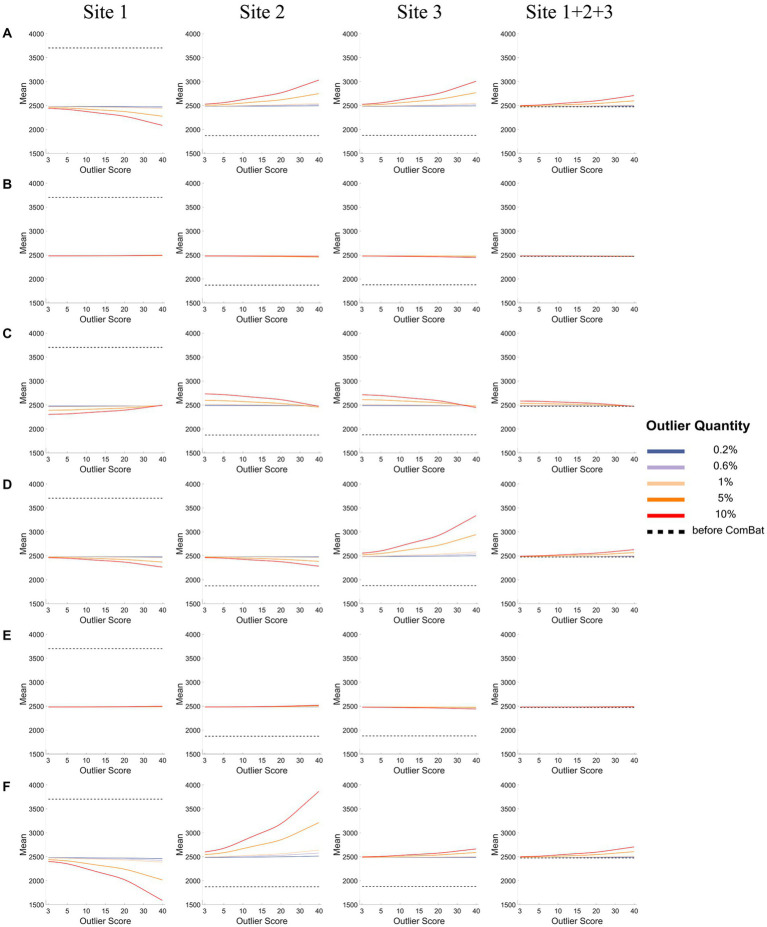
The mean of a representative IDP under different outlier scores and outlier quantities before and after ComBat harmonization. **(A-F)** represent the six outlier scenarios described in Section 2.2. The four columns represent the results of Site 1, Site 2, Site 3, and Site 1 + 2 + 3 (combined site), respectively. In each subgraph, the y-axis represents the mean value of the representative IDP (IDP-187), the x-axis represents the outlier scores, and different colors represent the outlier quantities (0.2, 0.6, 1, 5, 10%). Dashed lines in black represent the mean values before ComBat harmonization and colored solid lines represent the mean values after ComBat harmonization.

**Figure 5:**
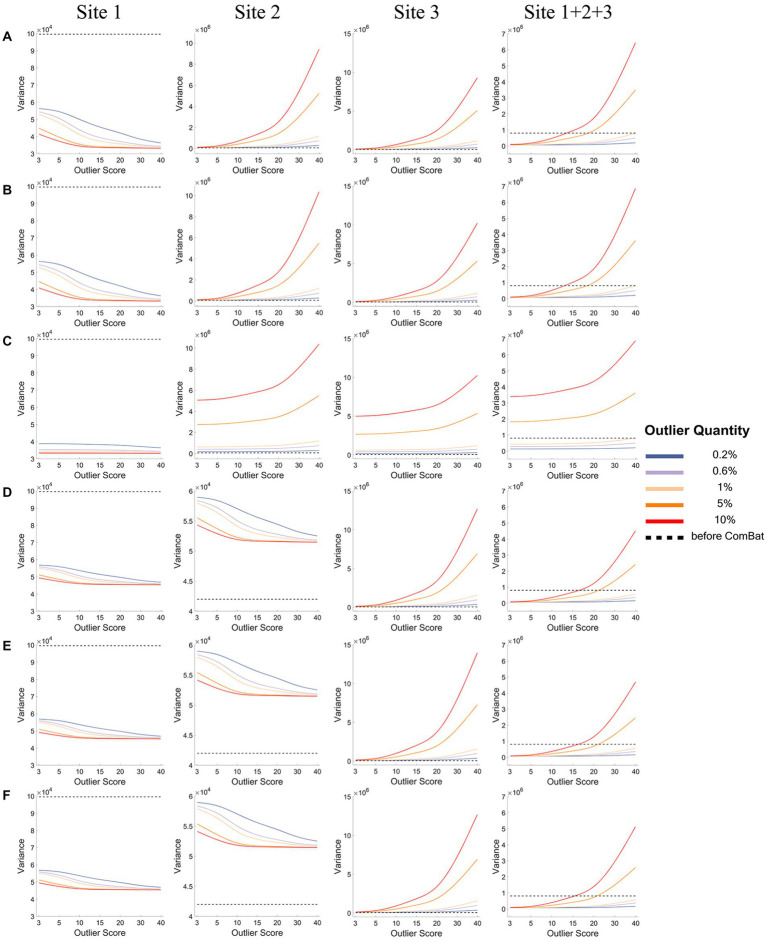
The variance of a representative IDP under different outlier scores and outlier quantities before and after ComBat harmonization. **(A-F)** represent the six outlier scenarios described in Section 2.2. The four columns represent the results of Site 1, Site 2, Site 3, and Site 1 + 2 + 3 (combined site), respectively. In each subgraph, the y-axis represents the variance of the representative IDP (IDP-187), the x-axis represents the outlier scores, and different colors represent the outlier quantities (0.2, 0.6, 1, 5, 10%). Dashed lines in black represent the variances before ComBat harmonization and colored solid lines represent the variances after ComBat harmonization.

Figures 4, 5 show how the mean (Figure 4) and the variance (Figure 5) of a representative IDP (IDP-187) of the normal samples (*y*-axis) changed with the outlier scores (x-axis) and with the outlier quantities (lines in different colors) both before ComBat harmonization (black dashed lines) and after ComBat harmonization (colored solid lines) in the six outlier scenarios for each site and the combined site.

As depicted in Figure 4, in all six outlier scenarios, there was a clear difference in the mean value of this representative IDP (IDP-187) between different sites before ComBat harmonization (black dashed lines), but such difference in the mean value was largely eliminated after ComBat harmonization (colored solid lines) unless the outlier quantity and the outlier score were high (outlier quantity≥5% and the outlier score ≥ 10). When the outliers were added to only one side in each site (panels A, D, and F) and when the outlier quantity and the outlier score were high (orange and red lines), we observed that the mean value was shifted towards the direction opposite to the side of outliers for the sites with outliers (i.e., Site 1 in panel A, and Sites 1&2 in panels D&F), but was shifted towards the same side of outliers or towards the side of more extreme outliers for the sites without outliers (i.e., Sites 2&3 in panel A and Site 3 in panels D&F). When the outliers were added to both sides of the data distribution in a balanced way (panels B&E), the mean value of each site (with or without outliers) did not change much with the outlier quantity and outlier score even when the outlier quantity and the outlier score were high. When the outliers were added to both sides in an unbalanced way and when the outlier quantity was high (orange and red lines for low outlier scores in panel C), the mean value was shifted towards the opposite side of more extreme outliers for the sites with outliers (i.e., Site 1 in panel C) but was shifted towards the same side of more extreme outliers for the sites without outliers (i.e., Sites 2&3 in panel C). As the shifts of mean values were in different directions in different sites, they canceled each out and resulted in a much less observable shift of the overall mean of the combined site.

As depicted in Figure 5, when the outliers were added to only one site (panels A-C), the data variance of the representative IDP (IDP-187) of the normal samples decreased greatly for the site with outliers (i.e., Site 1) after ComBat harmonization (colored solid lines) compared with before ComBat harmonization (black dashed lines), but opposite results were observed for the sites without outliers (i.e., Sites 2&3). Moreover, such changes in the data variance became bigger when the outlier quantity and outlier score increased. When the outliers were added to two sites (panels D-F), the same results were observed for the site with more extreme outliers (i.e., Site 1); however, the opposite results were observed for the site with less extreme outliers (i.e., Site 2), that is, the data variance of the normal samples increased for Site 2 after ComBat harmonization. It is also noticeable that, in all six outlier scenarios, the decrease of the variance in Site 1 (i.e., the site with more extreme outliers) was much larger than the increase of the variance in other sites (i.e., the site without outliers or the site with less extreme outliers) when the outlier quantity or the outlier score was low after ComBat harmonization compared with before ComBat harmonization, and thus the total variance of all data samples of the combined site (Site 1 + 2 + 3) showed a decrease after ComBat harmonization. In contrast, as the decrease of the variance in the sites with outliers was much lower than the increase of the variance in the sites without outliers when the outlier quantity or the outlier score was high after ComBat harmonization compared with before ComBat harmonization, the total variance of all data samples of the combined site showed an increase after ComBat harmonization. The reason for all these changes in data variance was because ComBat harmonization would equalize the data variance of different sites, and thus the distribution of the normal data in the site with very large outliers had to be squeezed and, at the same time, the distribution of the normal data in other sites had to be expanded so that the distributions of all data (including the outliers) in each site could have equal variance.

**Figure 6:**
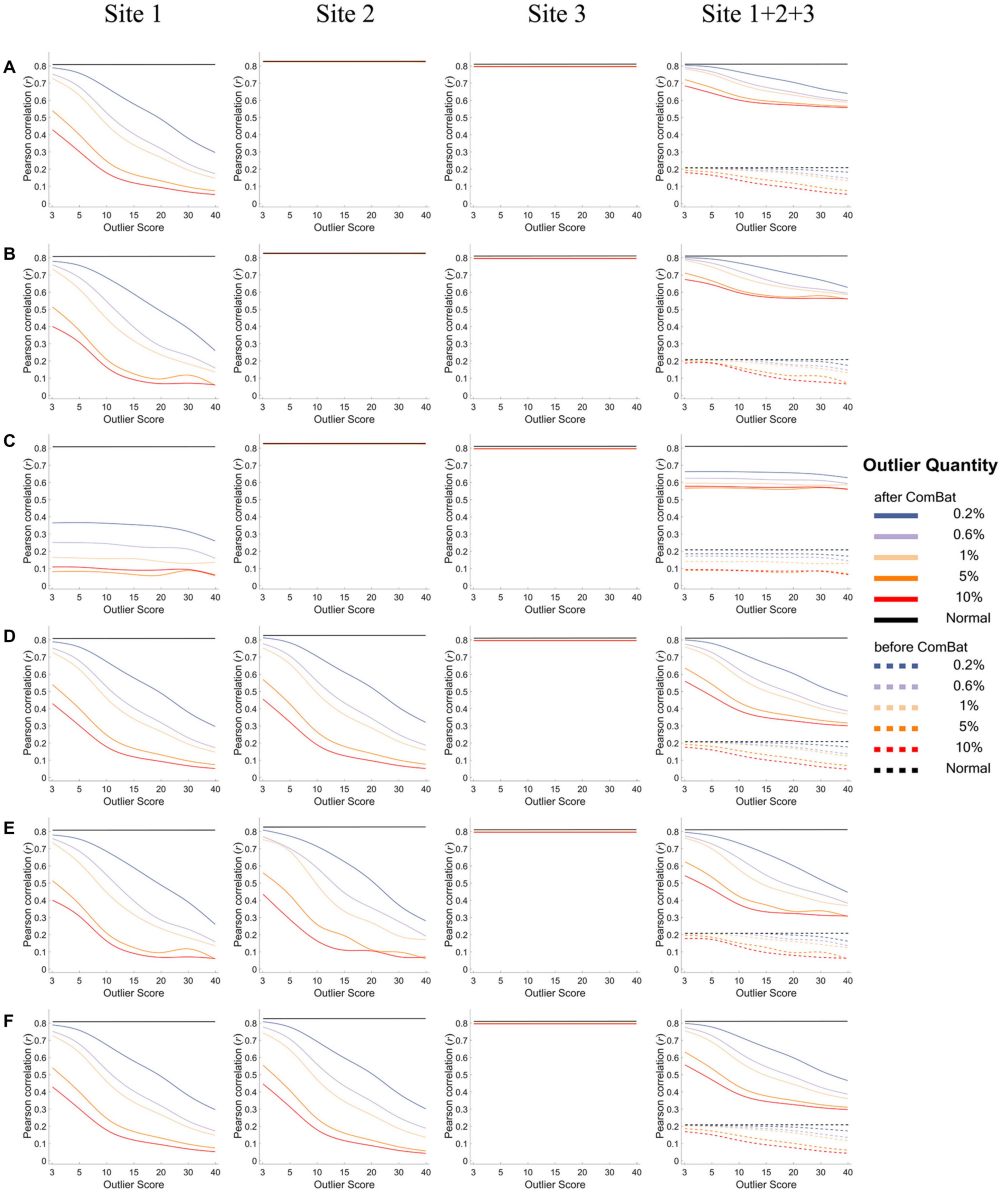
The IDP-behavior relationships measured by Pearson correlation coefficients under different outlier scores and outlier quantities before and after ComBat harmonization. **(A-F)** represent the six outlier scenarios described in Section 2.2. The four columns represent the results of Site 1, Site 2, Site 3, and Site 1 + 2 + 3 (combined site), respectively. In each subgraph, the y-axis represents the Pearson correlation coefficients (*r*) between the representative IDP (IDP-187) and the behavioral variable, the x-axis represents the outlier scores, and different colors represent the outlier quantities (0.2, 0.6, 1, 5, 10%, Normal) in which “Normal” means no outliers were added. Dashed lines represent the correlation coefficients before ComBat harmonization and solid lines represent the correlation coefficients after ComBat harmonization. Since within-site correlation coefficients before ComBat harmonization were the same as those after ComBat harmonization, dashed lines are overlapped with solid lines in Sites 1, 2, and 3.

**Figure 7:**
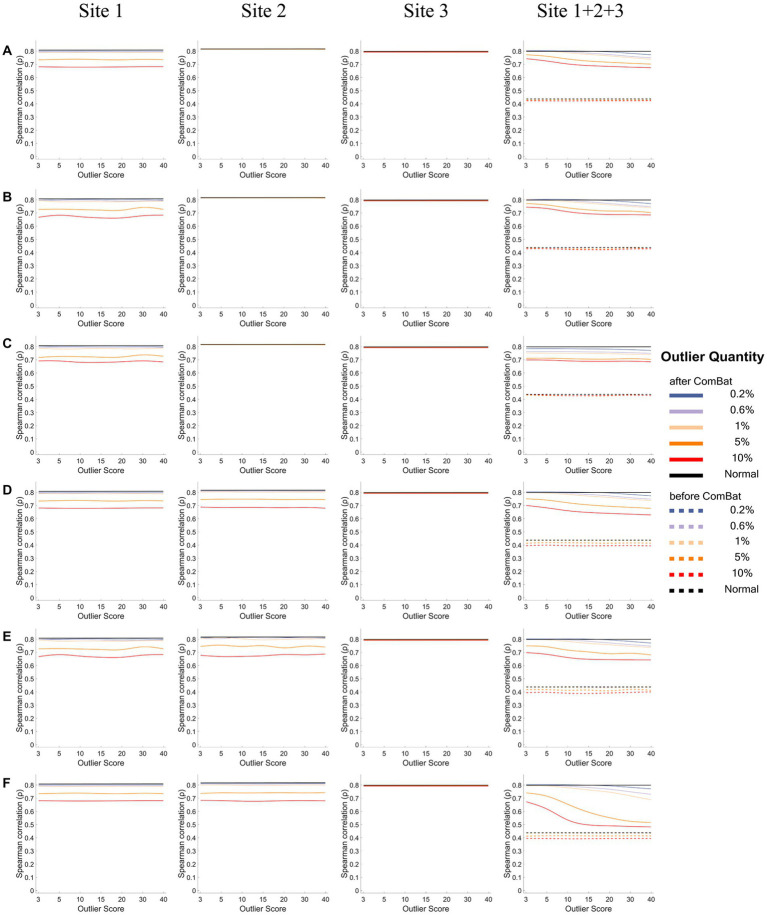
The IDP-behavior relationships measured by Spearman rank correlation coefficients under different outlier scores and outlier quantities before and after ComBat harmonization. **(A-F)** represent the six outlier scenarios described in Section 2.2. The four columns represent the results of Site 1, Site 2, Site 3, and Site 1 + 2 + 3 (combined site), respectively. In each subgraph, the y-axis represents the Spearman rank correlation coefficients (*ρ*) between the representative IDP (IDP-187) and the behavioral variable, the x-axis represents the outlier scores, and different colors represent the outlier quantities (0.2, 0.6, 1, 5, 10%, Normal) in which “Normal” means no outliers were added. Dashed lines represent the correlation coefficients before ComBat harmonization and solid lines represent the correlation coefficients after ComBat harmonization. Since within-site correlation coefficients before ComBat harmonization were the same as those after ComBat harmonization, dashed lines are overlapped with solid lines in Sites 1, 2, and 3.

Figures 6, 7 show how the IDP-behavior relationships (y-axis) changed with the outlier scores (x-axis) and with the outlier quantities (lines in different colors) for a representative IDP (IDP-187). The IDP-behavior relationships were measured by Person correlation coefficients (*r*) in Figure 6 and were measured by Spearman rank correlation coefficients (*ρ*) in Figure 7. The six panels (panels A-F in Figures 6, 7) show the results of the six outlier scenarios.

As depicted in Figure 6 (assessing IDP-behavior relationships using Pearson’s correlation coefficients), in all six outlier scenarios, all IDP-behavior correlation coefficients obtained before ComBat harmonization (dashed lines) were overlapped with those obtained after ComBat harmonization (solid lines) for within-site correlations (i.e., the left three columns), confirming that ComBat harmonization did not change the within-site IDP-behavior Pearson’s correlation coefficients. However, for a mega-analysis with cross-site correlations (i.e., the right column), the IDP-behavior Pearson’s correlation coefficients were very low before ComBat harmonization (dashed lines) but became much higher after ComBat harmonization (solid lines). In terms of the impacts of outlier scores and outlier quantities, the correlation coefficients remained the same for all outlier quantities and outlier scores in the sites without outliers (within-site correlations; Sites 2&3 in panels A-C and Site 3 in panels D-F) but decreased with the increase of the outlier quantity and outlier scores in the sites with outliers (within-site correlations; Site 1 in panels A-C and Site 1&2 in panels D-F) and in the combined site (across-site correlations; Site 1 + 2 + 3). In fact, compared with the true IDP-behavior correlation coefficient measured in the absence of outliers (~0.8; black lines), the correlation coefficient decreased to ~0.6 for “one-site-only” outlier scenarios (panels A-C) and to ~0.4 for “two-sites” outlier scenarios (panels D-F) when the outlier scores reached 15 and the outlier quantity reached 5% after ComBat harmonization in the combined site.

When using Spearman rank correlation to measure the IDP-behavior relationships (Figure 7), similar results were observed but with one exception: the Spearman correlation coefficients (*ρ*) did not change appreciably with the increase of the outlier scores in the sites with outliers for the within-site correlations (Site 1 in panels A-C and Site 1&2 in panels D-F).

As for the quantification of the effect of outlier location (i.e., outliers were unilateral or bilateral) on the IDP-behavior relationships, the ICCs of the 35 IDP-behavior correlation coefficients between the “unilateral” scenario and the “bilateral” scenario, calculated for the scenarios of “one-site outliers” (i.e., ICC was calculated between “one-site unilateral” and “one-site bilateral”) and for the scenarios of “two-site outliers” (i.e., ICC was calculated between “two-site unilateral,” “two-site bilateral” and “two-site different”) separately, are summarized in Table 1. It appeared that IDP-behavior relationships were not influenced much by outlier location – all ICCs were above 0.90 except for the ICC calculated based on the Spearman rank correlation coefficients for the scenarios of “two-site outliers” after ComBat harmonization (ICC = 0.77). Note that, the scenario of “one-site different” was not considered in this analysis because it was different from the other two “one-site” scenarios not only in outlier location but also in outlier score.

**Table 1:** ICCs of the 35 IDP-behavior correlation coefficients among different outlier scenarios.

		ICC (*r*)		ICC (*ρ*)	
		Figures 6A,B	Figures 6D–F	Figures 7A,B	Figures 7D–F
Before ComBat	Site 1	0.9951	0.9967	0.9876	0.9915
	Site 2	–	0.9968	–	0.9923
	combined data	0.9829	0.9842	0.9070	0.9951
After ComBat	Site 1	0.9951	0.9967	0.9876	0.9915
	Site 2	–	0.9968	–	0.9923
	combined data	0.9951	0.9981	0.9959	0.7723

*r* denotes Pearson correlation coefficient, *ρ* denotes Spearman rank correlation coefficient.

## 4. Discussion

In the present study, we tested whether and how the presence of outliers could affect the effectiveness of ComBat harmonization in the removal of inter-site heterogeneity using simulated multisite neuroimaging data. Importantly, the simulated dataset was generated based on a real large neuroimaging dataset, and different outlier properties such as the outlier location (i.e., where the outliers are, e.g., which sites, unilateral or bilateral), the outlier quantity (i.e., how many outliers are present) and the outlier score (how far the outliers deviate from the majority of the data) were modulated in the simulated data. With this design, we were able to test how these outlier properties could affect the effectiveness of ComBat harmonization using simulation data while maintaining a good representation of real data scenarios. We found that, although ComBat harmonization effectively removed the inter-site heterogeneity in multisite data which further improved greatly the detection of the true relationships between IDPs and behavioral variables in the absence of outliers, the presence of outliers could damage severely the performance of ComBat harmonization in the removal of data heterogeneity; instead, the presence of outliers might even introduce additional inter-site differences in the data. Such outlier effects were dependent on the quantity and deviation level of outliers – the effectiveness of ComBat harmonization was severely biased when the outlier quantity was greater than 5% or the outlier score exceeded 10; only moderate effects were observed otherwise. Moreover, we found that the degree of the impact of the outliers on the improvement of the detection of IDP-behavior relationships was dependent on how the IDP-behavior relationships were assessed (i.e., by Pearson correlation or Spearman correlation) – the degree of the impact was generally larger when Pearson correlation was used than when Spearman correlation was used, and was also dependent on outlier quantity and outlier score.

### 4.1. Presence of outliers reduces the effectiveness of ComBat harmonization in the removal of inter-site heterogeneity of data distribution

Although we have demonstrated the effectiveness of ComBat harmonization in the removal of inter-site heterogeneity in the absence of outliers, the results of our present study clearly showed that such effectiveness could be much reduced or even eliminated by the presence of outliers. We found that, after ComBat harmonization, the data distributions of the sites with outliers were no longer aligned with those of the sites without outliers in terms of the space covered by data samples (i.e., data variances); instead, the distribution of data majority became much denser for the sites with outliers. This is because the means and variances of multiple sites are supposed to be similar after ComBat harmonization, and the exaggerated variance caused by outliers would result in a decrease in the variance of normal values. In other words, due to the presence of outliers, the data variance would be overestimated and thus the variance of the data majority (i.e., the non-outlier samples) would shrink after ComBat harmonization even if there had been no differences in the mean or variance of non-outlier samples between this site and other sites before harmonization.

We also manipulated the location, the quantity, and the deviation level of outliers to test their impact on the data distribution (mean and variance of normal samples) of each site after ComBat harmonization. We found that the mean of normal samples was affected to an increasing extent, as the outlier quantity and outlier score increased. As for outlier location, when outliers were added to only one side of the data distribution, the mean of normal samples would shift opposite to where outliers were added. In the scenarios where outliers were added to both sides of the data distribution with different outlier scores, the mean of normal samples would shift opposite to where the outlier score was greater. We also found that the variances of normal samples decreased with the increase of outlier score and outlier quantity in the sites with outliers, resulting in a denser distribution of normal samples due to the existence of outliers.

### 4.2. Presence of outliers hampers the improvement of IDP-behavior correlation analyses by ComBat harmonization

As the presence of outliers reduces the effectiveness of ComBat harmonization in the removal of inter-site heterogeneity, they would thereby lead to erroneous assessment of IDP-behavior relationships, which were confirmed by the results of our present study. Furthermore, we found that the degree of the confounding effect of the outliers on IDP-behavior correlation coefficients was dependent on the outlier quantity and outlier score (Figures 6, 7) but was not affected much by the outlier location (Table 1). For across-site correlations in a multisite mega-analysis, the measurement of IDP-behavior relationships was affected by outlier quantities and outlier scores in all six outlier scenarios and for both correlation methods – both the Pearson correlation coefficients and the Spearman correlation coefficients decreased when the number of outliers and/or the outlier scores increased. However, it is worth noting that the impact of the outlier quantity and the outlier score on the correlation coefficients was greater for Pearson correlation than for Spearman correlation. This is because Spearman correlation utilized the rankings instead of original values to calculate correlation coefficients, which lessened the influence of extreme values on the estimation of correlation coefficients.

Although what we were mainly interested in here was the impacts of these outlier properties (i.e., quantity, outlier score, and location) on the assessment of across-site correlations between IDPs and behavioral variables in a mega-analysis after ComBat harmonization, we also examined the impacts on the within-site correlations to see whether and how ComBat harmonization could affect the assessment of IDP-behavior correlations in the presence of outliers within each site. For within-site correlations, the assessment of IDP-behavior relationships was unaffected for the sites without outliers which is expected given that it has been demonstrated using the normal simulation data in the present study that ComBat harmonization did not affect the correlation coefficients in the absence of outliers. However, the assessment of IDP-behavior relationships was affected by both outlier quantities and outlier scores for the sites with outliers in all six outlier scenarios especially when Pearson correlation was used – the Pearson correlation coefficients decreased gradually when the number of outliers and/or the outlier scores increased. Moreover, the impact of the outliers on the assessment of IDP-behavior relationships was also dependent on how such relationships were assessed. Pearson correlation coefficients were affected more badly than Spearman rank correlation coefficients. As the data samples were replaced with their rankings before the calculations of the correlation coefficient, the resultant correlation coefficient was more robust to the change of the actual IDP values caused by the change of outlier scores, although it still decreased with the increase of the number of outliers.

Compared with the impact of the outlier locations (i.e., outliers present unilaterally or bilaterally) on multisite data distribution, the IDP-behavior correlation coefficients measured either by Pearson correlation or Spearman rank correlation did not seem to be affected much by the outlier locations, except that the impact on the Spearman correlation coefficients seems smaller when the outliers were present only in one site than when they were present in two sites, especially when a relatively large number of outliers (≥5%) were added to different sides of data distribution on two sites. This is because a larger number of outliers, which was twice of the outliers added on single site, were added to two sites, and thus may disturb more badly the rankings and consequently the estimation of correlation coefficients.

Now that the presence of outliers could reduce the effectiveness of ComBat harmonization in the removal of inter-site heterogeneity of data distribution and in the detection of IDP-behavior correlations, we recommend, consistent with the workflow commonly used in most multisite studies, that outliers should be removed before ComBat harmonization. However, there might also be cases where outliers represent genuine biological variability (e.g., brain abnormalities due to diseases) and thus are of interest to be included in the study, it is possible to “harmonize” the outliers using the ComBat parameters generated from the data excluding outliers using the following procedure: remove the outliers first before ComBat harmonization, then estimate the batch-specific overall mean and variance parameters only using the normal data, and finally “harmonize” all data (including outliers) using the above-estimated parameters.

### 4.3. Limitations

There are a few limitations in the present study. First, although we were trying to be comprehensive by creating six outlier scenarios and modulating both outlier quantity and outlier score in each scenario, outliers in real data may be more complicated than all the scenarios simulated in the present study, and thus the impact of outliers on the effectiveness of ComBat harmonization may be more complex than what we demonstrated here. Second, we only focused on the ComBat harmonization method in the present study and thus how the presence of outliers affects the effectiveness of other harmonization methods remains unclear and needs to be addressed in the future. Third, we only demonstrated the effects of outliers on the effectiveness of ComBat harmonization using simulated data rather than real data because a meticulous quality control procedure was used during data acquisition of the CHIMGEN database and the quantity and deviation level of the outliers were very low in this particular real dataset (the outlier quantities were less than 0.64% and the outlier scores were less than 7.83 across all the IDPs), thus resulting in a minimal effect of outliers on the effectiveness of ComBat harmonization. Other real data with large and severe outliers may be tested for the effects of outliers on ComBat harmonization in future studies.

### 4.4. Conclusion

In summary, our present study systematically characterized the impact of outliers on the effectiveness of ComBat harmonization in the removal of inter-site heterogeneity of data distribution in multisite neuroimaging studies, and demonstrated that the presence of outliers may affect or even eliminate the effectiveness of ComBat harmonization and thus lead to erroneous results for the across-site analyses of brain-behavior associations. The findings deepen our understanding of the mechanisms by which outliers may affect the performance of ComBat harmonization and the results of subsequent brain-behavior association analyses according to their location, quantity, and deviation level, and highlight that the influence of outliers is not negligible and they must be detected and removed carefully prior to ComBat harmonization in multisite neuroimaging studies.

## Data availability statement

The simulation data supporting the conclusions of this article will be made available by the authors, without undue reservation.

## Author contributions

QH and ML designed the study. QH, XX, and SW analyzed the data. QH and XX visualized results and drafted this article. QH, XX, and ML revised this article. WQ, CY, and ML advised the design and data analysis of this study. All authors contributed to the article and approved the submitted version.

## Funding

This study was funded by the National Natural Science Foundation of China (Grant nos: 81971694 and 81971599).

## Conflict of interest

The authors declare that the research was conducted in the absence of any commercial or financial relationships that could be construed as a potential conflict of interest.

## Publisher’s note

All claims expressed in this article are solely those of the authors and do not necessarily represent those of their affiliated organizations, or those of the publisher, the editors and the reviewers. Any product that may be evaluated in this article, or claim that may be made by its manufacturer, is not guaranteed or endorsed by the publisher.
